# Pretreatment of Garlic Oil Extracts Hampers Epithelial Damage in Cell Culture Model of Peptic Ulcer Disease

**DOI:** 10.3390/medicina58010091

**Published:** 2022-01-07

**Authors:** Lucija Kuna, Milorad Zjalic, Tomislav Kizivat, Hrvoje Roguljic, Vjera Nincevic, Tea Omanovic Kolaric, Catherine H. Wu, Aleksandar Vcev, Martina Smolic, Robert Smolic

**Affiliations:** 1 Department of Pharmacology and Biochemistry, Faculty of Dental Medicine and Health Osijek, J. J. Strossmayer University of Osijek, 31000 Osijek, Croatia; lkuna@fdmz.hr (L.K.); hroguljic@mefos.hr (H.R.); vnincevic@fdmz.hr (V.N.); tomanovic@fdmz.hr (T.O.K.); 2 Department of Pharmacology, Faculty of Medicine Osijek, J. J. Strossmayer University of Osijek, 31000 Osijek, Croatia; 3 Department of Medical Biology and Genetics, Faculty of Medicine, J. J. Strossmayer University of Osijek, 31000 Osijek, Croatia; mzjalic@mefos.hr; 4 Department of Nuclear Medicine and Oncology, Faculty of Medicine Osijek, J. J. Strossmayer University of Osijek, 31000 Osijek, Croatia; tkizivat@mefos.hr; 5 Clinical Institute of Nuclear Medicine and Radiation Protection, University Hospital Osijek, 31000 Osijek, Croatia; 6 Department of Internal Medicine, University Hospital Osijek, 31000 Osijek, Croatia; avcev@fdmz.hr; 7 Department of Medicine, Division of Gastroenterology-Hepatology, University of Connecticut Health Center, Farmington, CT 06030, USA; cwu@uchc.edu; 8 Department of Pathophysiology and Physiology with Immunology, Faculty of Dental Medicine and Health Osijek, J. J. Strossmayer University of Osijek, 31000 Osijek, Croatia; 9 Department of Pathophysiology, Faculty of Medicine, J. J. Strossmayer University of Osijek, 31000 Osijek, Croatia

**Keywords:** peptic ulcer disease, sodium taurocholate, garlic oil extracts, lansoprazole, AGS cell line

## Abstract

*Background and Objectives:* Peptic ulcer disease is a chronic disease affecting up to 10% of the world’s population. Proton pump inhibitors, such as lansoprazole are the gold standard in the treatment of ulcer disease. However, various studies have shown the effectiveness of garlic oil extracts in the treatment of ulcer disease. A cellular model can be established in the human gastric cell line by sodium taurocholate. The aim of this study was to explore the effects of garlic oil extracts pretreatment and LPZ addition in the cell culture model of peptic ulcer disease by examining oxidative stress and F-actin distribution. *Materials and Methods:* Evaluation was performed by determination of glutathione and prostaglandin E2 concentrations by ELISA; human gastric cell line proliferation by cell counting; expression of ATP-binding cassette, sub-family G, member 2; nuclear factor kappa B subunit 2 by RT PCR; and F-actin cytoskeleton visualization by semi-quantification of Rhodamine Phalloidin stain. *Results:* Our results showed significant reduction of cell damage after sodium taurocholate incubation when the gastric cells were pretreated with lansoprazole (*p* < 0.001) and increasing concentrations of garlic oil extracts (*p* < 0.001). Pretreatment with lansoprazole and different concentrations of garlic oil extracts increased prostaglandin E2 and glutathione concentrations in the cell culture model of peptic ulcer disease (*p* < 0.001). Positive correlation of nuclear factor kappa B subunit 2 (*p* < 0.01) with lansoprazole and garlic oil extracts pretreatment was seen, while ATP-binding cassette, sub-family G, member 2 expression was not changed. Treatment with sodium taurocholate as oxidative stress on F actin structure was less pronounced, although the highest concentration of garlic oil extracts led to a statistically significant increase of total amount of F-actin (*p* < 0.001). *Conclusions:* Hence, pretreatment with garlic oil extracts had gastroprotective effect in the cell model of peptic ulcer disease. However, further experiments are needed to fully elucidate the mechanism of this protective role.

## 1. Introduction

Peptic ulcer disease (PUD) is a chronic disease affecting up to 10% of the population worldwide. It is characterized by defects to the inner lining of the gastrointestinal tract (GI) due to the secretion of pepsin or gastric acid. These defects extend through the gastric epithelial layer into the muscularis mucosa [1,2].

The etiology of PUD is not fully understood, but it is generally accepted that it results from impaired homeostasis of gastroprotective factors, such as the mucosal-bicarbonate barrier and prostaglandin secretion, as well as aggressive factors such as gastric acid, pepsin and *Helicobacter pylori* (*H. pylori*) infection [3], and consequently an increased production of mucosal proinflammatory cytokines interleukin-1β (IL-1β), tumor necrosis factor alpha (TNFα), interleukin-17 (IL-17) and interferon gamma (IFNγ). An increase in the production of chemokines, such as macrophage inflammatory protein 2 (MIP-2) and RANTES secreted by activated T cells are released at the site of inflammation, leading to the exacerbation of the ulcerative process [4,5].

In addition, recent studies have shown that the long term use of non-steroidal anti-inflammatory drugs (NSAID), smoking, a spicy diet, and stress factors have led to an exponential increase in the incidence of gastric ulceration [3].

Pharmacotherapy of PUD includes several groups of drugs, but proton pump inhibitors (PPI) and antibiotic therapy for *H. pylori* infection represent the gold standard. Although conventional regimens are effective and safe, side effects can limit their long-term clinical utility. On the other hand, studies have demonstrated that herbal medicines exhibit therapeutic benefit for gastric ulcer with fewer side effects [1,6]. Therefore, one could argue that conventional drugs in combination with herbal medicine may have a synergistic effect in the treatment of gastric ulcer and prevention of disease recurrence. Herbal extracts are one of the most significant sources of new drugs and to date they have shown promising results in the therapy of PUD [7].

Historically, the health benefit of garlic oil extracts (GE) is known. The mechanisms by which GE work in the treatment of gastric ulcer have been partially explained by its observed antioxidant effect by scavenging reactive oxygen species (ROS), inhibition of gastric acid secretion, lipoprotein oxidation and reduction of H (+)/K (+)-ATPase activity [8]. Moreover, it has been shown that the effectiveness of garlic plant extracts (*Allium sativum*) is comparable to the effectiveness of drugs such as omeprazole or cimetidine while long-term use should have fewer adverse effects [9].

Because bile reflux can lead to gastric lesions [10] we propose to generate a model of PUD by treating human adenocarcinoma gastric cell line (AGS) with sodium taurocholate (NaT), a bile salt.

To evaluate the effect of antioxidants in GE on the AGS cell line in vitro, we measured cell survival; intracellular reduced glutathione (GSH) level; and prostaglandin E2 (PGE2) level. The supply of GSH reduces cell damage resulting from oxidative stress; however, the loss of the GSH-dependent enzyme pathway and consequently reduced GSH levels, contributes to the progression and formation of numerous diseases [11,12]. Another significant gastroprotective mechanism involves prostaglandins (PGs) which play an important role in the acceleration of ulcer healing and epithelial cell proliferation. PGE2, which is produced and stored in the gastric and duodenal mucosa, plays a key role in gastric acid suppression and pepsin, gastric mucus and bicarbonate secretion [7,13].

Recently, it has been demonstrated that the relative expression level of nuclear factor kappa B subunit 2 (NFκB2) gene is decreased in gastric cancer as opposed to PUD which might suggest the inhibition of the NF-κB pathway during carcinogenesis. One of the possible genetic factors contributing to the development of PUD could be the change in the ATP-binding cassette, sub-family G, member 2 (ABCG2) gene. It was reported that the more intense the infection of ulcer disease, the higher the level of ABCG2 expression. In addition, it is not elucidated whether GE impacts ABCG2 expression in the AGS cell culture model of PUD [14]. On the other hand, one of the most significant effects of oxidative stress could be cytoskeletal disruption and changes in the F-actin distribution [15]. Moreover, to the best of our knowledge, the effect of NaT on the cell cytoskeleton have not yet been thoroughly investigated.

Cell cytoskeleton protein actin is composed of actin filaments. Actin plays a significant role in cell motility and force generation [16]. Considering that a significant number of membrane proteins’ function involves various cytokine receptors, channel proteins, or signaling proteins, the redistribution of F actin is an indirect measure of a cell’s ability to act on external stimuli [17]. In our model of PUD, this refers to the ability of AGS cells following external stimulation by NaT, LPZ, and GE. Furthermore, since the actin fibers are anchored internally to the cell membrane, a significant and permanent disruption of the membrane structure will lead to a disruption in the structure, distribution and amount of F actin.

Therefore, the aim of our study was to clarify whether (i) there a significant increase in PGE2 and GSH levels in a GE pretreated cell culture model of NaT-induced PUD that can be reflected in changes in cell number; (ii) whether GE pretreatment induces changes in ABCG2 and NFκB2 gene expression levels that would further elucidate the pathway by which GE might eventually reduce ulceration; and (iii) to characterize the effect of NaT on F-actin distribution.

## 2. Materials and Methods

### 2.1. Cell Culture

Human epithelial gastric cell line AGS (ATCC CRL-1739) has been used. The AGS cell line was derived from fragments of a tumor resected from a patient who had received no prior therapy. The AGS cells are mucus-secreting epithelial cells presenting numerous characteristics of well-differentiated gastric cells, including mucus production and morphology, and have been used as a model for testing of gastroprotective drug potentials [18]. Cells were cultured in 10 cm dishes in Roswell Park Memorial Institute medium (RPMI-1640), (Sigma-Aldrich, St. Louis, MO, USA) containing 2 mM L-glutamine supplemented with 10% fetal bovine serum (FBS), (Thermo Fisher Scientific Inc., Waltham, MA, USA) and 1% antibiotic/antimycotic solution (Thermo Fisher Scientific Inc., Waltham, MA, USA). Cells were grown at 37 °C in a humidified atmosphere of 5% CO2 (*v/v*) in air. Cells were passaged every 3–4 days to maintain 75% confluency.

### 2.2. Sodium Taurocholate-Induced Damage to AGS Cells

To determine NaT-induced damage, AGS cells were cultured as mentioned above. Cells were grown for 2 days to reach 90% confluence, and followed by exposure to increasing concentrations of NaT, (Sigma Aldrich, St. Louis, MO, USA) for 30 min for up to 24 h in triplicates, respectively. To establish NaT toxicity, cells were maintained in RPMI 1640 without FBS. The antiproliferative and cytotoxic effect was determined by a colorimetric MTT (3-(4, 5-dimethyl-2-thiazolyl) -2, 5-diphenyl -2H-tetrazolium bromide) (Sigma Aldrich, St. Louis, MO, USA) assay. The absorption was measured at 450 nm on a microplate reader (iMarkTM Microplate Absorbance Reader; Bio-Rad, Hercules, CA, USA) according to the manufacturer’s protocol and as described previously [19].

### 2.3. Measurement of the Gastroprotective Effect of Garlic Extracts (GE) in a Cell Culture Model of Peptic Ulcer Disease

The cell subgroups for determination of gastroprotective effect of GE were designed as follows: group A, AGS cells grown in RPMI-1640 medium as a negative control; group B, AGS cells treated only with LPZ (≥98% powder (TLC); Sigma Aldrich, MO, USA) (10 µM) as a positive control; group C, AGS cells treated with NaT (4 mM) only; group D, AGS cells pre-treated with LPZ (10 µM) and exposed to NaT (4 mM); groups E-H, cells pre-treated with increasing concentrations of GE and exposed to NaT (4 mM). To prevent oxidative stress, AGS cells (groups E–H) were pre-treated with 100, 150, 250 and 350 μg/mL concentrations of oil extracts of garlic (Sigma Aldrich, St. Louis, MO, USA). GE stock was prepared in 6% bovine serum albumin (BSA) in a 1:6 garlic oil to BSA ratio and homogenised with an ultrasonic homogenizer (Bandelin Sonoplus 2070) for 15 s. Cells were plated at a density of 4 × 10^5^ cells/mL in 6-well plates and were grown for 24 h. On the second day, cells were exposed to the GE in various concentrations for 24 h. On the third day, to induce oxidative stress in cells, the 4 mM NaT was added to the medium without FBS for 1 h. Subsequently, cells were trypsinized to determine cell viability using trypan blue exclusion and a Neubauer Hemocytometer used to cell numbers. Results were expressed as a percentage relative to negative controls of at least three independent experiments.

### 2.4. Measurement of Cellular Glutathione (GSH) Concentration

To determine the level of free radicals accumulation, the concentration of GSH was measured by ELISA. On the first day of experiment, cells were plated at a density of 4 × 10^5^ cells/mL of medium in 6-well plates. Eight subgroups were denoted as follows: untreated cells, cells treated with LPZ only, cells treated with NaT only, cells treated pre-treated with LPZ and subsequently exposed to NaT, and cells pre-treated with four different concentrations of GE and subsequently exposed to NaT. GSH concentration were determined using a commercially available Glutathione Colorimetric Detection Kit (Thermo Fisher Scientific Inc., Waltham, MA, USA) according to the manufacturer’s protocol and as briefly described by our group earlier [20]. The response was measured using an iMarkTM Microplate Absorbance Reader at 405 nm. Results were expressed as micromoles per milliliter per well.

### 2.5. Measurement of Prostaglandin E2 (PGE2) Concentration

To determine PGE2 concentration, the day after the AGS cells become confluent, eight subgroups were denoted as follows: untreated cells, cells treated with LPZ only, cells treated with NaT only, cells pre-treated with LPZ and subsequently exposed to NaT, and cells pre-treated with four different concentrations of GE and subsequently exposed to NaT. The PGE2 content was determined by an enzyme immunoassay kit (Elabscience, Houston, TX, USA) as described by the manufacturer. Briefly, after incubation, cells were washed with pre-cooled phosphate buffered saline (PBS) and dissociated by trypsin. Cells were collected into the tubes and centrifuged for 5 min at 1000× *g*. Medium was discarded and cell pellets were washed 3 times with pre-cooled PBS. The freeze–thaw process was repeated several times, and centrifuged for 10 min at 1500× *g* at 4°. The cell fragments were removed, and supernatant was collected to carry out the assay. The response was measured using an iMarkTM Microplate Absorbance Reader at 450 nm. The values were calculated according to the manufacturer’s instructions. Results were expressed in picograms per milliliter per well.

### 2.6. Total RNA Isolation and Reverse Transcription Polymerase Chain Reaction (RT-PCR) Analysis

To evaluate the expression of NFκB2 and ABCG2, total RNA was isolated on day three of the experiment using the RNeasy Mini Kit (Qiagen, Hilden, Germany) according to the manufacturer’s protocol. First strand cDNA was synthesized by manufacturer’s protocol (PrimeScript First StrandcDNASynthesis Kit, Takara Bio, Otshu-Shi, Japan). The synthesized cDNA was amplified using specific primer sequences as follows: β actin (sense 5′-GCACCACACCTTCTACAATG-3′, antisense 5′-TGCTTGCTGATCCACATCTG-3′); NFκB2 (sense 5′-CCATGACAGCAAATCTCC-3′, antisense 5′-TAAACTTCATCTCCACCC-3′); ABCG2 (sense 5′-ATGTCAACTCCTCCTTCTAC-3 ′antisense 5′AATGATCTGAGCTATAGAGGC-3′). PCR conditions were: for β actin denaturation at 94 °C for 3 min, annealing at 56.7 °C for 45 s, elongation at 72 °C for 1 min in 30 cycles; for NFκB2 denaturation at 94 °C for 3 min, annealing at 50 °C for 45 s, elongation at 72 °C for 1 min in 30 cycles; for ABCG2 denaturation at 94 °C for 3 min, annealing at 54 °C for 45 s, elongation at 72 °C for 1 min in 30 cycles. The PCR products were run on 0.8% agarose gel, stained with SYBR Safe DNA Gel Stain (Thermo Fischer Scientific, Waltham, MA, USA), visualized and semi quantified by ImageJ software using QuantIF ImageJ macro [21].

### 2.7. Visualization of the F-Actin Cytoskelet with Rhodamine Phalloidin Stain

To determine the organization and structure/function relationships of filamentous structures in a cell model of PUD, the AGS cells were grown on glass cover-slips inside a six well plate grown to 85% confluency in RPMI-1640 plus 10% FBS. After 24 h, cells were treated as follows: untreated cells (grown in RPMI-1640 medium), cells treated with LPZ only, cells treated with NaT only, cells pretreated with LPZ and subsequently exposed to NaT and cells pretreated with four different concentrations of GE and subsequently exposed to NaT. On the third day, according to the above described protocol, cells were exposed to 4 mM NaT for 1 h. AGS cell morphology (F-actin cytoskelet) was visualized by Rhodamine Phalloidin Reagent (Abcam Inc., Cambridge, UK) according to the manufacturer’s instructions. Briefly, cell culture medium was aspirated carefully to avoid dislodging of any cells from the plate. After that, cells were washed once in PBS and fixed in 3–4% formaldehyde in PBS at room temperature for 10–30 min. 0.1% Triton X-100 (Haihang Industry Co., Ltd, Jinan, China) in PBS was added into the fixed cells for 3–5 min. Cells were washed 2–3 times in PBS. Conjugate working solution, 100 µL of 1× Phalloidin was added to each well of fixed cells. Cells were incubated in the dark, at room temperature for 60 min. Nuclei were counterstained using 4,6-diamidino-2-phenylindole (DAPI) (1 µg/mL in methanol). Subsequently, cells were rinsed 2–3 times with PBS. The cells were analyzed using an AxioSkop 2 MOT microscope (Car Zeiss, Göttingen, Germany) equipped with fluorescence and Zeiss filter sets 15 and 01. Cell images were obtained by using a 40× dry immersion objective adjusted against stain negative control with an Olympus DP70 camera (American laboratory trading, Inc., East Lime, CT, USA) and without post-acquisition enhancement of images. Total and immuno-positive nuclei were counted in ImageJ software using QuantIF ImageJ macro [21].

### 2.8. Statistical Analyses

For statistical significance data was analyzed with One way and Two way ANOVA post hoc Tukey HSD. Data eligibility for analysis with ANOVA was determined with Shapiro Wilk test for normality of distribution and with Bartlett’s F test for homoscedasticity on samples. *p* values of * *p* < 0.05, ** *p* < 0.01 and *** *p* < 0.001 were considered statistically significant.

## 3. Results

### 3.1. Establishment of the Cell Culture Model of Peptic Ulcer Disease and Assessment of the Toxic Effect of Sodium Taurocholate (NaT)

**Figure 1:**
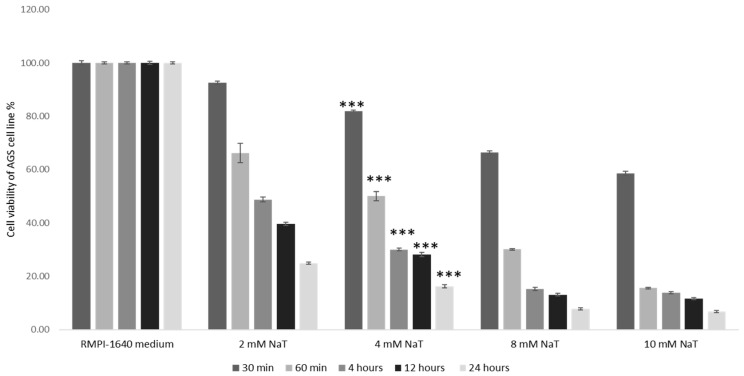
Establishment of the cell culture model of peptic ulcer disease and assessment of the toxic effect of NaT. Determination of cell viability by MTT assay after exposure to varying NaT concentrations and varying time periods in AGS cell line. Two way ANOVA _F(125,149)_ = 1.18 × 10^4^; *p* = 3.73 × 10^−160^ post hoc Tukey HSD. Bars assigned with asterisks are statistically significantly different (*** *p* < 0.001) compared to untreated control. The data shown are representative of at least three independent experiments. Sodium taurocholate (NaT), human gastric cell line (AGS).

The toxicity of the NaT on the AGS cell viability were assessed by MTT assay after treatment with four different doses of NaT: 2, 4, 8 and 10 mM, and at different time periods: 30 min, 1, 4, 12 and 24 h, respectively. Each experiment was repeated at least three times to ensure consistency of the results. After exposure to 4 m NaT for 60 min, cell had 50% viability compared to untreated control (*p* < 0.001) as determined by MTT assay shown in Figure 1. According to the MTT results, the concentration and length of exposure to NaT were selected. The results showed that reduction of cell viability by 50% required 4 mM NaT after exposure for 1 hour, and this concentration and exposure time were used in all subsequent experiments on the AGS model of PUD.

### 3.2. Gastroprotective Effect of Garlic Extracts (GE) in a Cell Culture Model of Peptic Ulcer Disease

**Figure 2:**
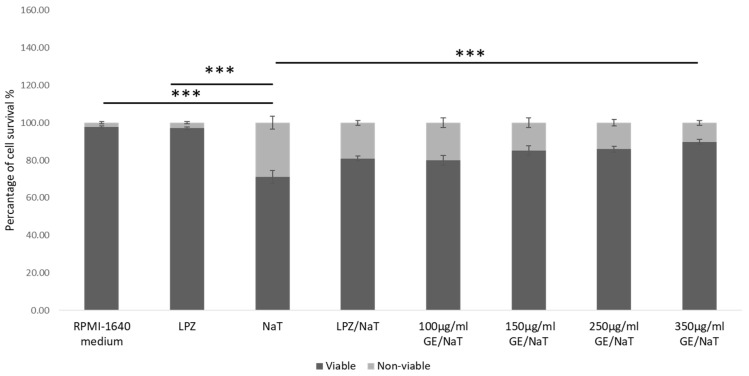
Effects of GE on the proliferation and apoptosis of AGS cell line. One way ANOVA _F(7,23)_ = 19.78; *p* = 9.26 × 10^−7^; post hoc Tukey HSD. The values are represented as means ± SD. Bars assigned with asterisks are statistically significantly different (*** *p* < 0.001). The data shown are representative of at least three independent experiments. Human gastric cell line (AGS), sodium taurocholate (NaT/4 mM), lansoprazole (LPZ/10 µM), garlic extracts (GE/100, 150, 250 and 350 µg/mL).

To determine effects of GE on cell survival, cells were pretreated with GE in four different concentrations for 24 h, as shown in Figure 2. On the second day the medium without FBS was changed and cells were exposed to 4 mM NaT for 1 h. Cell survival was determined, and compared to untreated control and cells treated with LPZ only. In AGS cells, GE at concentrations 100, 150, 250 and 350 µg/mL showed statistically significant higher survival compared to NaT-alone treated cells (*p* < 0.001). Survival of NaT treated cells was 64%, while survival of 350 µg/mL GE pretreated cells was 94% (*p* < 0.001). There was no significant difference between cells pretreated with LPZ and subsequently with NaT (cell survival was 80%) and with cells pretreated with highest concentration of GE subsequently exposed to NaT (cell survival was 89%). Further, there was no statistically significant difference between highest concentration of GE compared to untreated cells and cells treated with LPZ only.

### 3.3. Measurement of Cellular Glutathione (GSH) Concentration in a Cell Culture Model of Peptic Ulcer Disease

**Figure 3:**
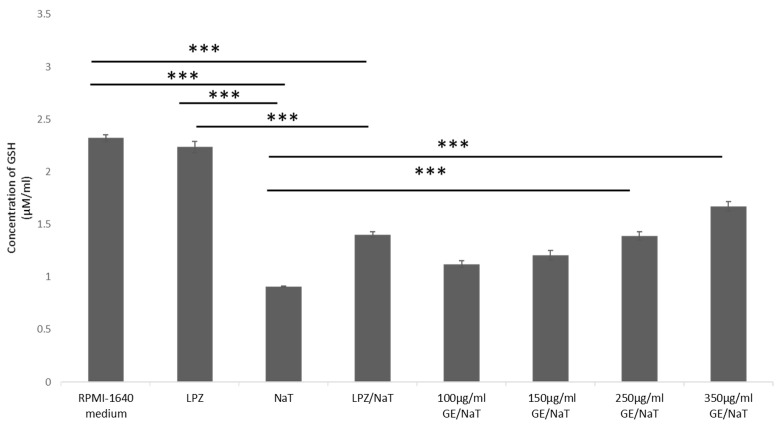
Effects of GE pretreatment on the levels of GSH in AGS cell line. GSH measurements were carried out by spectrophotometry at 415 nm. One way ANOVA _F(7,23)_ = 72.99; *p* = 5.99 × 10^−11^; post hoc Tukey HSD. The values are represented in micromole per milliliter as average with standard deviation ± SD. Bars assigned with asterisks are statistically significantly different (*** *p* < 0.001) The data shown are representative of at least three independent experiments. Cellular glutathione (GSH), human gastric cell line (AGS), sodium taurocholate (NaT/4 mM), lansoprazole (LPZ/10 µM), garlic extracts (GE/100, 150, 250 and 350 µg/mL).

To evaluate cellular redox tone, GSH levels were measured in the above mentioned subgroups, as shown in Figure 3. Untreated cells (RPMI 1640 medium), and cells inoculated with LPZ were used as a controls. In AGS cell line, treatment with NaT only showed significant decrease of GSH levels compared to untreated control and LPZ treatment only (*p* < 0.001). Pretreatment with 250 and 350 µg/mL of GE caused significant recovery of GSH levels compared to cells treated with NaT only (*p* < 0.001). Moreover, there was significant difference in GSH levels between cells pretreated with LPZ and subsequently with NaT and cells treated with LPZ only (*p* < 0.001), shown in Figure 3.

### 3.4. Measurement of Prostaglandin E2 (PGE2) Concentration in a Cell Culture Model of Peptic Ulcer Disease

**Figure 4:**
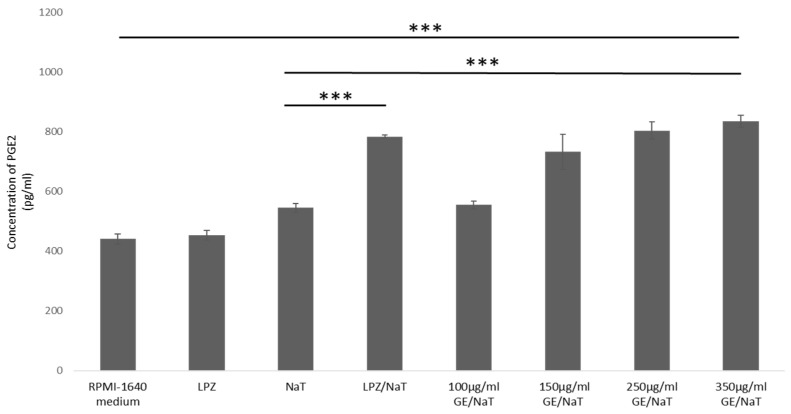
Effects of GE pretreatment on the levels of PGE2 in AGS cell line. PGE2 measurements were carried out by spectrophotometry at 450 nm. One way ANOVA _F(7,23)_ = 187.1; *p* = 3.98 × 10^−14^; post hoc Tukey HSD. The values are represented in picograms per milliliter as average with standard deviation ± SD. Bars assigned with asterisks are statistically significantly different (*** *p* < 0.001) The data shown are representative of at least three independent experiments. Prostaglandin E2 (PGE2), human gastric cell line (AGS), sodium taurocholate (NaT/4 mM), lansoprazole (LPZ/10 µM), garlic extracts (GE/100, 150, 250 and 350 µg/mL).

To evaluate the role of prostaglandin in AGS model of ulcer disease, PGE2 concentrations as a powerful stimulus of gastric mucus were measured. Pretreatment with GE at concentrations 100, 150, 250 and 350 µg/mL stimulated PGE2 synthesis compared with untreated control and LPZ only (*p* < 0.001). The GE at 350 µg/mL presented a strong cytoprotective effect in AGS cell line increasing the levels of PGE2 compared with NaT solely. However, in AGS cell line, treatment with NaT showed significant increase of PGE2 levels compared to untreated control and LPZ only (*p* < 0.001), as shown in Figure 4. There was no significant difference in PGE2 synthesis between GE at 100 µg/mL and cells treated only with NaT.

### 3.5. Visualization and Quantification of the F-Actin Cytoskeleton with Rhodamine Phalloidin Stain in a Cell Culture Model of Peptic Ulcer Disease

Deformations and distribution of the F-actin cytoskeleton are involved in the epithelial cells’ damage. To assess the effect of ulcerogenic agent on epithelial gastric cells, distribution of F-actin was measured. 

**Figure 5:**
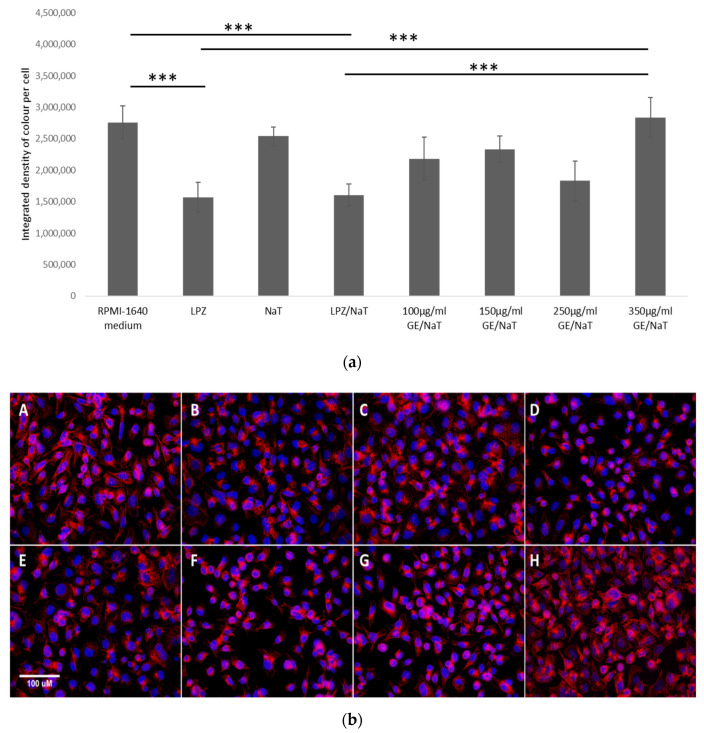
Quantification and Visualization of the F-actin cytoskelet with Rhodamine Phalloidin stain. (**a**) Levels of total F actin stained by Rhodamine Phalloidin treated with LPZ, NaT and GE in AGS cell line. Data represent integrated density of red color of stained actin per single cell. Higher number equals more intense stain. One-way ANOVA _F(7,32)_ = 18.47, *p* = 1.37 × 10^−9^. The values are represented as means ± SD. Bars assigned with asterisks are statistically significantly different (*** *p* < 0.001) The data shown are representative of at least three independent experiments. Sodium taurocholate (NaT/4 mM), lansoprazole (LPZ/10 µM), garlic extracts (GE/100, 150, 250 and 350 µg/mL). (**b**) AGS cells were labeled for F-actin using Rhodamine Phalloidin and nuclei stained with DAPI. A—RPMI 1640 (control), B—lansoprazole (LPZ 10 µm), C—sodium taurocholate (NaT/4 mM), D—sodium taurocholate (NaT 4 mM) and lansoprazole (LPZ 10 µM), E—sodium taurocholate (NaT 4 mM) and garlic extracts (GE 100 µg/mL), F—sodium taurocholate (NaT 4 mM) and garlic extracts (GE 150 µg/mL), G—sodium taurocholate (4 mM) and garlic extracts (GE 250 µg/mL), H—sodium taurocholate (4 mM) and garlic extracts (GE 350 µg/mL). Size bar represents 100 µm.

In both cases, AGS cells treated with LPZ only and cells pretreated with LPZ and subsequent with NaT caused a significant decrease in the total amount of actin within the cell (*p* < 0.001). On the other hand, treatment with NaT only did not show decrease in the total amount of actin compared to the control group. At the same time, pretreatment with GE exposed to NaT in the highest concentration at 350 µg/mL did not show statistically significant reduction in distribution of F-actin in the total amount of actin compared to untreated control. However, there was significant difference between the highest concentration of GE compared to cells pretreated with LPZ and subsequent exposed to NaT (*p* < 0.001), as shown in Figure 5a,b.

### 3.6. Expression of ABCG2 and NFκB2 in a Cell Culture Model of Peptic Ulcer Disease

**Figure 6:**
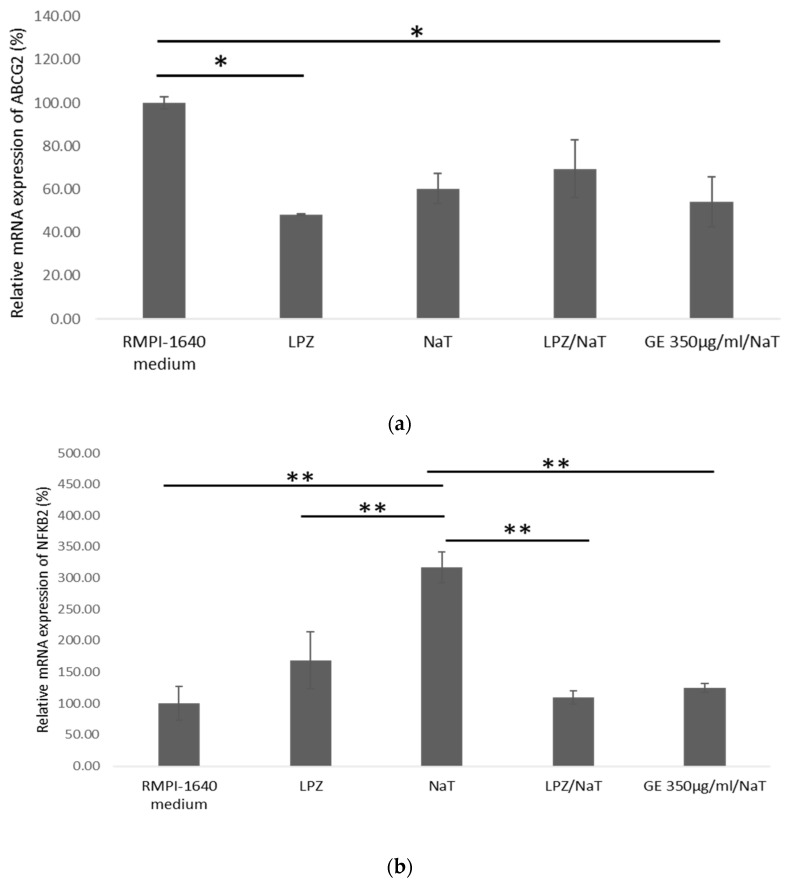

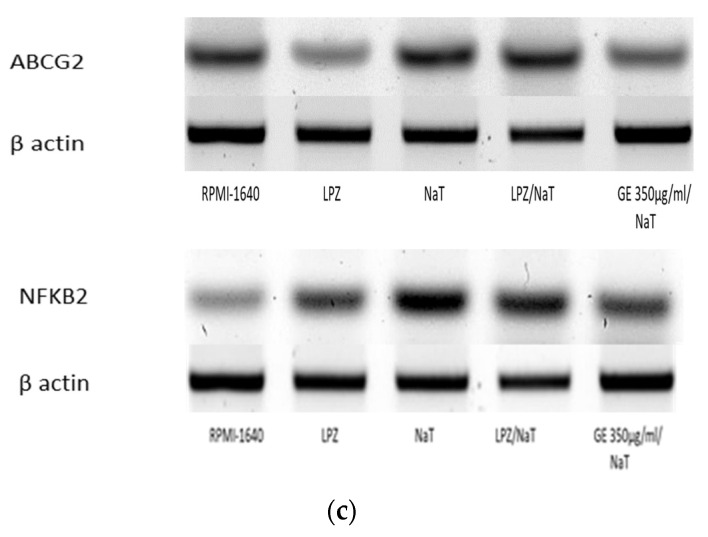
Expression of ABCG2 and NFκB2 in cell culture model of peptic ulcer disease. (**a**) ABCG2 gene expression in AGS treated cell line. One-way ANOVA _F(4,14)_ = 5.683 *p* = 0.01191; post hoc Tukey HSD test; * *p* < 0.05. The gene expression analysis was carried out by RT-PCR and obtained results were semi-quantified ImageJ software using QuantIF ImageJ macro. The values are represented as means ±SD. The data shown are representative of three independent experiments. ATP-binding cassette sub-family G, member 2 (ABCG2). (**b**) NFκB2 gene expression in AGS treated cell line. One-way ANOVA F_(4,14)_ = 11.61, *p* = 0.0008926; post hoc Tukey HSD test; ** *p* < 0.01. The gene expression analysis was done by RT-PCR and obtained results were semi-quantified by ImageJ software using QuantIF ImageJ macro. The values are represented as means ± SD. The data shown are representative of three independent experiments. Nuclear Factor Kappa B Subunit 2 (NFκB2). (**c**) Representative figures of Southern blot analysis of ABCG2 and NFκB2 expression compared to β actin expression.

To further evaluate possible genetic factors contributing to the development of PUD, changes in the ABCG2 gene expression was measured. ABCG2 expression was decreased in cells pretreated with the highest concentration of GE subsequently exposed to NaT, and cells pretreated with LPZ only compared to untreated control (*p* < 0.05). However, cells treated only with NaT showed lower expression of ABCG2 compared to untreated cells, as shown in Figure 6a. There were no other statistically significant differences between groups.

NF-κB is encoded by NFκB2, activated by inflammatory factors such as IL-8, and infection during the development of peptic ulcer phosphorylation and degradation of l kB through I kB kinase complex. To assess the possible genetic factors significant for the development of PUD, relative expression level of the NFκB2 gene was investigated. Expression of NFκB2 at highest concentration of GE exposed to NaT was significantly lower compared to cells treated with NaT only (*p* < 0.01), as shown in Figure 6b. Strong expression of NFκB2 in cells treated with NaT only was also shown compared to untreated cells, cells treated with LPZ only and cells pretreated with LPZ subsequently exposed to NaT. (*p* < 0.01). However, there was no statistically significant difference between cells pretreated with LPZ and subsequent exposed to NaT, and cells pretreated with highest concentration of GE subsequent exposed to NaT.

## 4. Discussion

Examination of various plant extracts has led to the discovery of new pharmacological ingredients with effective gastroprotective activity. For instance, antioxidant properties of GE are used as the main mechanism for the treatment of PUD (63). In the last few years, a number of in vivo studies have shown the antiproliferative and antioxidant activity effects of several compounds derived from GE. These include flavonoids, flavanols, polyphenols, and sulfur-containing compounds, known as the main compounds involved in its bioactivity, which play significant role in scavenging free radicals [8,22].

However, the potential gastroprotective effect of GE in in vitro studies especially on the gastric human cell line has not yet been fully elucidated. Previous studies have shown that a model of gastric cells impaired by NaT was used to explore the gastroprotective effect of different ingredients against bile-induced injury on the gastric mucosa [10,23]. Hence, in our study the model of AGS damaged by NaT was used to determine the gastroprotective effect of GE in culture model of PUD. Treatment with 4 mM NaT for 60 min caused a reduction of 50.03% in cell viability compared to untreated controls. 

A pretreatment of 24 h with increasing concentrations of GE showed cytoprotective effect on the cell damage caused subsequently by NaT; cell survival in cells pretreated with 250 and 350 µg/mL of GE and subsequently exposed to NaT increased significantly compared to cells exposed to NaT only. These results indicate that cytoprotective compounds of garlic have significant role in protection of AGS cells against NaT-induced damage binding bile salts, consequently forming a barrier to avoid injury to mucous membranes. Our results can be compared with a previous investigation which demonstrated antioxidant dose dependent effect in reducing oxidative stress [20]. However, pretreatment of GE at increasing concentrations was similar to cells pretreated with LPZ and subsequently with NaT. Further, no difference was present in the highest concentration of GE compared to LPZ and untreated control. Therefore, from these results, we can conclude that GE could act to promote ulcer healing and prevent recurrence.

Previous in vitro and in vivo studies have shown that intracellular GSH protects gastric mucosal cells against ethanol-induced damage [3,24]. In a study from Rodriguez at al., terpenes and their derivatives have been shown to have gastroprotective activity and produce a significant rise of GSH concentration in AGS cells [25,26]. The results of Rodriguez at al. were confirmed by our current study in which the concentration of GSH increased, after the cells were pretreated with GE and subsequently damaged by NaT. Our data on GSH levels clearly showed that NaT damage increased oxidative stress, while pretreatment with GE resulted in a beneficial effect on reducing oxidative stress. However, the pretreatment with the highest concentration of GE followed by exposure to NaT showed higher levels of GSH in comparison to cells pretreated with LPZ and then with NaT. This could indicate the strong role of garlic as a natural antioxidant against oxidative damage. Moreover, in a study performed by Stagos et al., quercetin, a well-known plant polyphenol found in GE, significantly increased GSH level and inhibited the reduction of GSH both in vivo and in vitro [27]. In a study from Ashmawy at al., aged garlic extracts decreased oxidative stress caused by indometacin-gastic ulcer and led to reduced depletion of GSH [28]. Therefore, GSH activity of GE provides further evidence to support usage of GE in prevention of PUD. 

Prostaglandin E2 is powerful inhibitor of gastric acid secretion and stimulus of gastric mucus and bicarbonate synthesis. In our study, NaT did not show a significant effect on increased secretion of PGE2 compared to LPZ or untreated control, while in combination with LPZ prostaglandin secretion was increased. This would suggest that our model of PUD is a very complex inflammatory process involving different mediators of inflammation; however, damage caused with NaT solely did not show the most significant role in PGE2 synthesis. Moreover, in a previous study on rat models, a single dose of LPZ did not affect the synthesis of PG [29]. Omeprazole also failed to affect PG production from gastric mucosal cells [30]. However, GE significantly stimulated PGE2 production, while the greatest effect was found in its highest concentration. Previous research done by De Olinda et al. reported that *Magonia glabrata* was demonstrated to protect against gastric lesions caused by indomethacin and ethanol by increasing production of PG [31]. Likewise, results of our study are in agreement with the study of Theoduloz at al., which showed how derivates of the medicinal plant *Jatropha isabelii* affect increases of PGE2 synthesis after damage with bile salt [10]. It is known that garlic contains fatty acids responsible for increasing level of PG which modulates inflammation. Moreover, several studies have showed that flavonoids (abundant in garlic) increased the gastric PGE2 level in an ethanol and acetic-induced gastric model in mice [32,33]. Hence, these studies suggest a significant role of medicinal plants in increasing PG production, but more studies are needed to specify the mechanisms by which garlic stimulates PG synthesis.

Actin cytoskeleton is a dynamic, fibrous network which is regulated by the coordinated action of actin-binding proteins. Previous studies had shown that deformations of the actin cytoskeleton are involved in the epithelial cell damage induced by taurocholate, ethanol and acetylsalicylic acid. However, ROS seems to underlie ethanol, but not acetylsalicylic acid or taurocholate, induced cytoskeletal disruption [34]. Quite unexpectedly, in our study treatment of NaT alone did not cause reduction of F-actin distribution, while LPZ causes significant decline. Hence, we can only assume that the reduced distribution of F actin with the addition of LPZ is the result of actin filaments’ reorganization. On the other hand, treatment with NaT as oxidative stress on F actin structure is less pronounced. Our data are comparable with a previous study on rat gastric mucosal cells which showed that exposure to 1–5 mmol/L taurocholate did not induce any changes in the actin bundles, while actins were moderately damaged by 10 mmol/L of taurocholate [34].

In our study the highest concentration of GE led to a statistically significant total amount of F-actin compared to LPZ treatment alone and to a combination of NaT and LPZ treatment. Gruhkle at al. showed in their study that the cytoplasmic branches of the actin cytoskeleton are lost in allicin-treated cells. Moreover, the actin filaments become amorphous, which indicated that allicin, found in garlic extract, has strong beneficial effect on the actin cytoskeleton [15]. Therefore, the results from our study support the protective role of GE in AGS cells damaged with NaT. Notwithstanding, proteins of cytoskeleton are most abundantly S-thio-allylated proteins, but the mechanism of actin cytoskeleton disruption has to be further investigated.

Protein product of the ABCG2 gene is found in various gastrointestinal tissues. However, any changes in the ABCG2 gene, may affect the protein level and its function [14]. Previous research reported that *H. pylori* in PUD could increase the ABCG2 gene expression [35]. In contrast, in our model significant downregulation of ABCG2 expression was observed in cells treated with NaT only, cells pretreated with the GE and subsequently exposed to NaT, and cells pretreated with LPZ. On the other hand, a higher expression of ABCG2 in cells treated with RPMI 1640 medium was demonstrated, thus indicating that the differences in the modulatory effects of NaT and GE on ABCG2 gene expression could be related to the different experimental paradigms used. Likewise, previous research has shown that the level of ABCG2 expression differed highly between numerous studies and cases [14]. Moreover, as ABCG2 protein was not found in the epithelium of the stomach, Diestra at al. reported that ABCG2 gene expression could derive from the capillaries’ endothelial cells, where expression of the protein was demonstrated [36]. Hence, it could be speculated that expression of ABCG2 gene in the model of PUD could be under the influence of NaT. Considering this and previous findings, the decreased expression of ABCG2 in the model of ulcer disease caused by NaT damage remains unanswered.

NF-κB plays a significant role in relief complications related to numerous chronic diseases and stress conditions. The sulfur compounds present in aged garlic extracts are known as key regulators of the inflammatory response, acting by reducing NF-κB activation, consequently preventing the production of proinflammatory cytokines such as interleukin 1 (IL-1) and interleukin 6 (IL-6). Several studies have also shown that NF-κB is activated by inflammatory factors such as IL-8 and *H. pylori* during development of PUD [26,37]. Moreover, Geng et al. showed that Jurkat T cells treated with S-allyl-cysteine (SAC), a garlic component, were able to prevent the NF-κB activation, while Schäfer and his colleagues reported on the ability of the garlic components SAC, allicin and diallyl-disulfide to inhibit activation of NF-κB [38,39].

In agreement, our data showed that GE inhibited NF-κB activation. Obviously, garlic derivatives have strong influence on the NF-κB regulatory pathway under stress conditions. Zebrowska at al. showed that the relative expression level of NF-κB is decreased in gastric cancer as opposed to PUD [40]. In our current study, there was a significant upregulation of NFκB2 expression in cells treated with NaT compared to other subgroups, indicating that NaT increases cellular damage and could have a significant role in development of PUD.

Finally, it is important to mention the effect of GE, especially its sulfur compounds, in other pathologies. Organic sulfur compounds, such as allin, allicin, ajoene, allyl-propyl disulfide, diallyl trisulfide (DATS), SAC and vinyl-dithiines are major active ingredients in garlic [41]. SAC, a water soluble organosulfur compound, has demonstrated suppressive effect of *H. pylori* on gastric inflammation in vivo and reduction of gastric cancer in a clinical trial [42]. Organosulfur compounds have the ability to inhibit the proliferation of human cancer cells, and to induce apoptosis in tumor cells in numerous tissues [43]. Additionally, SAC has a key role as endogenous donor of H2S in the liver and GI system. Its ability to reduce the expression of transaminase levels in tetrachloride (CCl)-induced liver fibrosis in rats [44] has also been demonstrated. In a study by Broncaccio at al., ovothiol, known as a natural product which includes sulfur-containing compounds, showed antifibrotic effect reducing fibro-genic markers involved in the progression of liver fibrosis, such as α-smooth muscle actin (α-SMA), transforming growth factor (TGF-β) and tissue metalloproteinase inhibitor (TIMP-1) [45]. It has also been demonstrated that garlic organo-sulfur compounds allin, allicin and S-allyl-cysteine inhibited transglutaminase (tTG) activity, a well-known biomarker for liver fibrosis. Moreover, in in vitro experiments, despite its lower concentration, GE has shown anti-fibrotic effect in liver homogenate to the same extent as cysteine, known as specific tTG inhibitors [46]. Additionally, in a study performed by D’Argenio at al., treatment with GE reduced CCl-induced liver fibrosis leading to reduction of oxidative and endoplasmic reticulum stress and regeneration of the liver through the transforming growth factor beta 1 (TGF-β1) signaling pathway [44,47].

## 5. Conclusions

In conclusion, GE exhibit anti-ulcer protective mechanisms, such as cytoprotective effects and antioxidant activity, increasing concentrations of GSH and PGE2. Our results demonstrated that the attributed GE gastroprotective effects against NaT-PUD occur due to oxidative stress via blockade of pro-inflammatory signaling mediated by the NF-κB pathway. ABCG2 gene expression had no connection with development of PUD, while the mechanism of F-actin cytoskeleton disruption in our model has to be further investigated. Overall, this research suggests that garlic has a significant role for further study as preventive agent for PUD. However, further pharmacological studies in other experimental models are necessary to elucidate the gastroprotective role of GE in PUD.

## Data Availability

The data presented in this study are available on request from the corresponding authors.

## Abbreviations

ABCG2	ATP-binding cassette, sub-family G, member 2
AGS	Adenocarcinoma epithelial gastric cell line
BSA	Bovine serum albumin
CCl	Tetrachloride
DAPI	4,6-diamidino-2-phenylindole
DATS	Diallyl trisulfide
FBS	Fetal bovine serum
GE	Garlic oil extracts
GI	Gastrointestinal
GSH	Glutathione
*H. pylori*	*Helicobacter pylori*
IFN-γ	Interferon gamma
IL-17	Interleukin 17
IL-1	Interleukin 1
IL-6	Interleukin 6
LPZ	Lansoprazole
MIP-1	Macrophage Inflammatory Protein 2
MTT	3-(4, 5-dimethyl-2-thiazolyl) -2, 5-diphenyl-2H- tetrazolium bromide
NaT	Sodium taurocholate
NFκB2	Nuclear factor kappa B subunit 2
NSAID	Non-steroidal anti-inflammatory drugs
PBS	Phosphate buffered saline
PG	Prostaglandin
PGE2	Prostaglandin E2
PPI	Proton pump inhibitors
PUD	Peptic ulcer disease
RANTES	Regulated upon Activation, Normal T Cell Expressed and Presumably Secreted
ROS	Reactive oxygen species
SAC	S-allyl-cysteine
SOD	Superoxide dismutases
TNF-α	Tumor necrosis factor alpha
TGF-β1	Transforming growth factor beta 1
tTG	Transglutaminase
TIMP-1	Tissue metalloproteinase inhibitor
SAC	S-allyl-cysteine
α-SMA	α-smooth muscle actin
